# Twenty Years of DENV-2 Activity in Brazil: Molecular Characterization and Phylogeny of Strains Isolated from 1990 to 2010

**DOI:** 10.1371/journal.pntd.0002095

**Published:** 2013-03-14

**Authors:** Nieli Rodrigues da Costa Faria, Rita Maria Ribeiro Nogueira, Ana Maria Bispo de Filippis, Jaqueline Bastos Santos Simões, Fernanda de Bruycker Nogueira, Monique da Rocha Queiroz Lima, Flavia Barreto dos Santos

**Affiliations:** Flavivirus Laboratory, Oswaldo Cruz Institute, Manguinhos, Rio de Janeiro, Rio de Janeiro, Brazil; Centers for Disease Control and Prevention, United States of America

## Abstract

In Brazil, dengue has been a major public health problem since its introduction in the 1980s. Phylogenetic studies constitute a valuable tool to monitor the introduction and spread of viruses as well as to predict the potential epidemiological consequences of such events. Aiming to perform the molecular characterization and phylogenetic analysis of DENV-2 during twenty years of viral activity in the country, viral strains isolated from patients presenting different disease manifestations (*n* = 34), representing six states of the country, from 1990 to 2010, were sequenced. Partial genome sequencing (genes C/prM/M/E) was performed in 25 DENV-2 strains and full-length genome sequencing (coding region) was performed in 9 strains. The percentage of similarity among the DENV-2 strains in this study and reference strains available in Genbank identified two groups epidemiologically distinct: one represented by strains isolated from 1990 to 2003 and one from strains isolated from 2007 to 2010. No consistent differences were observed on the E gene from strains isolated from cases with different clinical manifestations analyzed, suggesting that if the disease severity has a genetic origin, it is not only due to the differences observed on the E gene. The results obtained by the DENV-2 full-length genome sequencing did not point out consistent differences related to a more severe disease either. The analysis based on the partial and/or complete genome sequencing has characterized the Brazilian DENV-2 strains as belonging to the Southeast Asian genotype, however a distinction of two Lineages within this genotype has been identified. It was established that strains circulating prior DENV-2 emergence (1990–2003) belong to Southeast Asian genotype, Lineage I and strains isolated after DENV-2 emergence in 2007 belong to Southeast Asian genotype, Lineage II. Furthermore, all DENV-2 strains analyzed presented an asparagine (N) in E_390_, previously identified as a probable genetic marker of virulence observed in DHF strains from Asian origin. The percentage of identity of the latter with the Dominican Republic strain isolated in 2001 combined to the percentage of divergence with the strains first introduced in the country in the 1990s suggests that those viruses did not evolve locally but were due to a new viral Lineage introduction in the country from the Caribbean.

## Author Summary

In Brazil, the first dengue haemorrhagic cases were reported after the DENV-2 introduction in Rio de Janeiro, which spread to other states in the country. Aiming to perform the molecular characterization and phylogenetic analysis of DENV-2 during twenty years of viral activity in the country, strains isolated from patients presenting different disease manifestations were sequenced. Phylogeny characterized the DENV-2 as belonging to the Southeast Asian genotype, however a distinction of two Lineages within this genotype has been identified. Furthermore, all strains presented an asparagine in E_390_, previously identified as a probable genetic marker of virulence. The results show a temporal circulation of genetically different viruses in Brazil, probably due to the introduction of a new viral lineage from the Caribbean, which lead to the re-emergence of this serotype after 2007, causing the most severe epidemic already described in the country.

## Introduction

Dengue viruses (DENV) are the most important human arboviruses worldwide, transmitted by mosquitoes of the genus *Aedes*, *Aedes aegypti* is the main vector. Explosive epidemics have become a public health problem, economic impact, socially and politically significant [1], [2].

Currently it is estimated that 70 to 500 millions dengue infections occur annually in 124 endemic countries. Nearly 3.6 billion people (55% of world population) are at risk of contracting the disease (DVI). The rapid global spread of DENV in the last 50 years resulted in the dispersal of genotypes associated with increased severity [3].

The four serotypes (DENV-1, DENV-2, DENV-3 and DENV-4) are closely related yet antigenically distinct and contain a positive-sense RNA genome that is translated as a single polyprotein and post-translationally cleaved into three structural proteins, capsid (C), premembrane (prM) and envelope (E), and seven nonstructural proteins, NS1, NS2A, NS2B, NS3, NS4A, NS4B and NS5. The RNA genome is packaged in an icosahedral capsid, and the nucleocapsid is surrounded by a lipid bilayer containing the E and M proteins [4], [5].

DENV infection causes a spectrum of clinical disease ranging from an acute debilitating, self-limited febrile illness - dengue fever (DF) - to a life-threatening syndrome - dengue hemorrhagic fever/dengue shock syndrome (DHF/DSS) [6]. Despite the similar disease manifestations, the DENV are genetically diverse with approximately 40% of amino acid sequence divergence. Distinct DENV genotypes can be characterized when the genetic divergence are higher than to 6% [7].

A recent analysis of 1,827 complete E gene sequences supported the existence of six genotypes for DENV-2: Asian genotype I, Asian genotype II, Southeast Asian/American genotype, Cosmopolitan genotype, American genotype and the Sylvatic genotype, the most genetically distinct genotype. Furthermore, the Southeast Asian/American genotype's topologies suggested a spatial division of this genotype into two major subclades [8].

In the Americas, the first DHF epidemics in the 80's were due to the introduction of the Southeast Asian/American genotype which replaced the American genotype and more severe cases with higher viremia were reported [9]–[11].

In Brazil, the disease has become a public health problem with explosive epidemics after the introduction of DENV-1 in 1986 in Rio de Janeiro [12]. However, the first DHF/DSS cases were only reported after the DENV-2 introduction in 1990 in the country [13], [14]. From 1990 until the 26th epidemiological week of 2010, a total of 5,481,921 cases, including 17,203 cases of dengue hemorrhagic fever (DHF) and 1954 deaths were reported in the country [15].

Aiming to perform the phylogeny of the DENV-2 and its impact in the disease severity during 20 years of viral activity in Brazil, strains isolated from DF, DHF/DSS and fatal cases occurred since its introduction in 1990 until 2010, were analyzed. In this scenario, the partial sequencing (C/prM/M/E genes) of 25 DENV-2 strains was performed. To determine whether the evolutionary relationships observed for the C/prM/M/E genes were applicable to the complete genome, we further fully sequenced the coding regions of nine DENV-2 strains. In order to avoid mutations introduced by *in vitro* passages of the virus in cell cultures we used DENV-2 strains extracted directly from serum or originally isolated from cell cultures.

## Materials and Methods

### Ethical statement

The strains analyzed in this study belong to a previously-gathered collection from the Laboratory of Flavivirus, IOC/FIOCRUZ, Rio de Janeiro, Brazil, obtained from human serum through the passive surveillance system performed by the Laboratory from an ongoing Project approved by resolution number CSN196/96 from the Oswaldo Cruz Foundation Ethical Committee in Research (CEP 274/05), Ministry of Health-Brazil. Samples were chosen anonymously, based on the laboratorial results and clinical manifestations input on the Laboratory database.

### Viral strains

Viral strains consisted of DENV- 2 (*n* = 34) isolated during epidemics occurred from 1990 to 2010 in six states in Brazil (Table 1). Each sample was accompanied by identification form containing clinical and epidemiological data. All strains were determined as DENV-2 serotype by reverse transcriptase polymerase chain reaction (RT-PCR) and or/virus isolation from DF (*n* = 19), DHF (*n* = 3), DSS (*n* = 1) and fatal cases (*n* = 4; 1 from DF, 2 from DHF and 1 with no classification available). Seven cases were not classified due to data unavailability.

**Table 1:** Primers used for amplification of the partial and complete genes (coding region) from Brazilian DENV-2.

Primers Designation	Sense A (5′→3′)	Anti-sense B (5′→3′)	Position in the genome (according to AF489932)	Amplicon (pb)	Tm (°C) A/B
1	CGT GGA CCG ACA AAG ACA GA	GGA GCG ACG GCT GTC AGT AA	14–906	892	62/64
2	GAT CAG TGG CAC TCG TTC CA	CTC CGG GTAGCCATGGTAAC	708–1586	878	62/62
3	ATG GCA CTG TCA CGA TGG AG	CAC TAT CAG CCTGCACCATAGCT	1467–2405	938	62/63
4	GGA TCC CTG GGA GGA GTG TT	TCC ATT GCT CCA GAG GGT GT	2202–3106	904	63/63
5	GAC TCA AAA CTC ATG TCA GCG G	GTG CTT TGG GAA AGG AGT GC	2958–3800	842	62/62
6	GGG CGT TAC CAT GAC GGA T	GCC CAT GAT GGT TCA ATC CTT	3656–4709	1053	63/63
7	AAT TAC GGC AGC AGC ATG GT	GGA GGA GTG GCT GTC ATG AAA	4475–5456	981	63/63
8	CAG CCA TCA GAA CCG AGC A	CCA CCT TCT GTC TGC GTA GTT G	5254–6185	931	64/62
9	ACA CAC CTG AAG GAA TCA TTCCTA G	TGA CAA ATG TTG TAG CCA CGG	6016–6948	932	62/62
10	AGC CAT CCT CAC AGT GGT GG	TCT CAG TTT TGC TGA GCC TCG	6791–7737	946	64/63
11	CTA TTT GGC CGG AGC TGG A	TTT CAA TTC CAA TGT TGC GG	7508–8354	846	63/62
12	ATG GAG GAG CTT TAG TGA GGA ATC	CGT GCT CCA AGC CAC ATG TA	8170–8994	824	61/63
13	GAA ATC GGC TCG TGA GGC T	TCA TCT TGG TTT CTG CAT GGG	8825–9746	921	63/63
14	GAC AGT CAC AGA AGA AAT CGC TGT	CTA TGG CTT GAT CCG ACC TGA	9473–10304	831	62/62
15	CGG CTC ATT GAT TGG GCT AA	TTC TGT GCC TGG AAT GAT GCT	10109–10662	553	63/63

### RNA extraction

Viral RNA was extracted from infected cell culture supernatant or directly from the patients' serum using QIAamp Viral RNA Mini kit (Qiagen) following the manufacturer's instructions and stored at −70°C for DENV typing and sequencing.

### RT –PCR (Reverse transcriptase- polymerase chain reaction)

RT—PCR for detecting and typing DENV was performed as described previously [16]. Briefly, consensus primers were used to anneal to any of the four DENV types and amplify a 511-bp product in a reverse transcriptase-polymerase reaction. A cDNA copy of a portion of the viral genome was produced in a reverse transcriptase reaction. After a second round of amplification (nested PCR) with type-specific primers, DNA products of unique size for DENV-2 (119 bp) were generated.

### Dengue virus isolation

Virus isolation was performed by inoculation into C6/36 *Aedes albopictus* cell line [17] and isolates were identified by indirect fluorescent antibody test (IFAT) using serotype-specific monoclonal antibodies [18]. Briefly, patients' sera were inoculated into C6/36 *Aedes albopictus* cell monolayers in L-15 Medium (Leibovitz, Sigma) supplemented with 2% fetal calf serum (FCS, Invitrogen) and 0.2 mM of nonessential amino acids (Invitrogen). Cells were incubated at 28°C for 5 to 7 days and observed for cytopathic effects. Infected supernatant was clarified by centrifugation and virus stocks stored in 1-mL aliquots at −70°C until use.

### Sequencing

Reverse transcription (RT) was performed using 5 µL of extracted RNA in 25 µL of AccessQuick RT-PCR System (Promega Corporation) and specific oligonucleotides primers (Table 1). To amplify the C/prM/M/E region of 2,325 bp, specific primers (1 to 4) were used to produce 4 overlapping amplicons of approximately 900 bp and to amplify the complete coding region (10,173 bp), 15 overlapping amplicons of approximately 900 bp (1 to 15). Thermocycling conditions consisted of a single step of 42°C for 60 minutes and 40 cycles of denaturation at 94°C (30 seconds), annealing at 56°or 63°C (60 seconds) depending on the set of primers, extension at 72°C (2 minutes) and a final extension at 72°C (10 minutes). Amplification was conducted using a Model 9700 thermal cycler (Applied Biosystems). PCR products were purified from 1.0% agarose gels using QIAquick Gel extraction Kit or QIAquick PCR purification Kit (Qiagen) and used as template for cycle sequencing. Sequencing reactions were performed as recommended in the BigDye Dideoxy Terminator sequencing kit (Applied Biosystems) and the products were analyzed using an automated 3130 DNA Sequencer (Applied Biosystems). Partial sequences (C/prM/M/E) and complete coding sequences for the unprocessed polyprotein (5′ and 3′ noncoding regions excluded) were deposited in GenBank (Table 2).

**Table 2:** DENV-2 used in this study for partial (*n* = 25) and complete coding region (*n* = 9) sequencing.

Strain #	Year of isolation	State of origin	Clinical manifestation	Imunne response	Age	Gender	Sequence region	Acess number GenBank
44298	1991	BA	DF	S	NA	Fem	C/prM/M/E	HQ012508
48578	1994	CE	NA	ND	NA	Male	C/prM/M/E	HQ012509
51222	1995	RJ	NA	ND	NA	Fem	C/prM/M/E	HQ012510
52477	1995	RJ	NA	ND	NA	Fem	C/prM/M/E	HQ012511
55769	1996	RS*	DF	ND	10	Male	C/prM/M/E	HQ012512
55803	1996	BA	NA	S	NA	Fem	C/prM/M/E	HQ012513
58448	1997	RN	DF	ND	NA	Male	C/prM/M/E	HQ012514
59382	1997	RN	DHF/Fatal	ND	NA	Male	C/prM/M/E	HQ012515
63291	1998	RJ	DF	ND	16	Male	C/prM/M/E	HQ012516
64625	1999	RJ	DF	ND	34	Male	C/prM/M/E	HQ012517
66985	2000	RJ	DF	ND	39	Male	C/prM/M/E	HQ012518
67955	2000	RJ	DHF	ND	27	Male	C/prM/M/E	HQ012519
69221	2001	RJ	DF	ND	28	Male	C/prM/M/E	HQ012520
72308	2001	RJ	DF	ND	62	Fem	C/prM/M/E	HQ012521
75103	2002	RJ	DF	ND	61	Masc	C/prM/M/E	HQ012522
76012	2002	ES	NA	ND	41	Fem	C/prM/M/E	HQ012523
77395	2003	ES	NA	ND	50	Male	C/prM/M/E	HQ012524
86977	2007	RJ	DHF	ND	7	Male	C/prM/M/E	HQ012525
88034	2007	RJ	DF	ND	12	Male	C/prM/M/E	HQ012526
0030	2008	RJ	DF	S	13	Male	C/prM/M/E	HQ012527
0832	2008	RJ	DHF	S	8	Fem	C/prM/M/E	HQ012528
066	2009	BA	DF	ND	1 month	Male	C/prM/M/E	HQ012529
0145	2009	ES	DF	ND	16	Male	C/prM/M/E	HQ012530
023	2010	RJ	DF	ND	73	Male	C/prM/M/E	HQ012531
0199	2010	RJ	DSS	S	50	Fem	C/prM/M/E	HQ012532
39145	1990	RJ	DF	ND	41	Fem	Complete CR	HQ012538
41768	1990	RJ	DF	ND	10	Male	Complete CR	HQ012533
42727	1991	RJ	DF	P	NI	Fem	Complete CR	HQ012534
48622	1994	CE	NA	ND	NI	Fem	Complete CR	HQ012535
61310	1998	RJ	DF	ND	47	Fem	Complete CR	HQ012536
64905	1999	RJ	DF	ND	52	Fem	Complete CR	HQ012537
0337	2008	RJ	Fatal	S	5 days	NA	Complete CR	NA
0450	2008	RJ	DF/Fatal	S	46	Male	Complete CR	NA
0690	2008	RJ	DHF/Fatal	S	32	Male	Complete CR	HQ026763

BA: Bahia, CE: Ceará, RJ: Rio de Janeiro, RS: Rio Grande do Sul, RN: Rio Grande do Norte, ES: Espírito Santo; DF: Dengue Fever; DHF: Dengue Hemorrhagic Fever; DSS: Dengue Shock Syndrome; Fem: Female; Male; C/prM/M/E: Capsid/pré-membrane/Membrane/Envelope; Complete CR: Complete coding region;

* Imported case; NA: Not available; ND: Not done; P: primary infection; S: secondary infection.

### Sequences and phylogenetic analysis

The analysis of similarities, percentage of identity and divergence among the strains analyzed were performed using Megalin Program (DNAstar, www.dnastar.com). The multiple alignment was performed using CLUSTAL W (http://www.ebi.ac.uk/clustalw/) and the phylogenetic analysis by MEGA 4 software (www.megasoftware.net), using the Maximum Likelihood method (ML), according to the Tamura-Nei model, with a bootstrap of 1,000 replications. Strains representative from the five genotypes available in Genbank (www.ncbi.nlm.nih.gov) were used for the comparison, DENV-1 (GenBank accession number GU370049), DENV-3 (accession number EF629369), and DENV- 4 (accession number AF289029) strains were used as outgroup to root the trees (Table 3).

**Table 3:** Strains representative of the different DENV-2 genotypes and strains used as outgroup for comparison purposes.

Strain #	Year of isolation	Country	Genotype	GenBank Accession #
BR64022	1998	Brazil	Southeast Asia (Lineage I)	AF489932
BID-V3496	1990	Venezuela	Southeast Asia (Lineage I)	GQ868540
N.1409	1983	Jamaica	Southeast Asia (Lineage I)	M20558
BID-V2683	1999	Nicaragua	Southeast Asia (Lineage II)	GQ199895
BID-V2996	2007	Nicaragua	Southeast Asia (Lineage II)	GQ199868
BID-V595	2006	Puerto Rico	Southeast Asia (Lineage II)	EU482726
BID-V1439	2005	Puerto Rico	Southeast Asia (Lineage II)	EU687216
DR23/01	2001	Dominican Republic	Southeast Asia (Lineage II)	AB122020
DR59/01	2001	Dominican Republic	Southeast Asia (Lineage II)	AB122022
BID-V3653	2008	Brazil	Southeast Asia (Lineage II))	GU131885
China-04	1985	China	Asian II	AF119661
New Guinea C	1944	New Guinea	Asian II	AF038403
Strain 44	1989	China	Asian II	AF204177
TB16i	2004	Indonesia	Asian I	AY858036
98900666 DSS DV-2	1998	Indonesia	Asian I	AB189124
IQT1797	1995	Peru	American	AF100467
strain 131	1992	Mexico	American	AF100469
isolate 1328	1977	Puerto Rico	American	EU056812
Dak Ar D75505	1991	Senegal	Sylvatic	EF457904
DENV-1-SGEHI(D1)1494Y08	2008	Singapore	-	GU370049
BRDEN3 290-02	2002	Brazil	-	EF629369
DENV-4-Guangzhou B5	2000	China	-	AF289029

## Results

**Figure 1:**
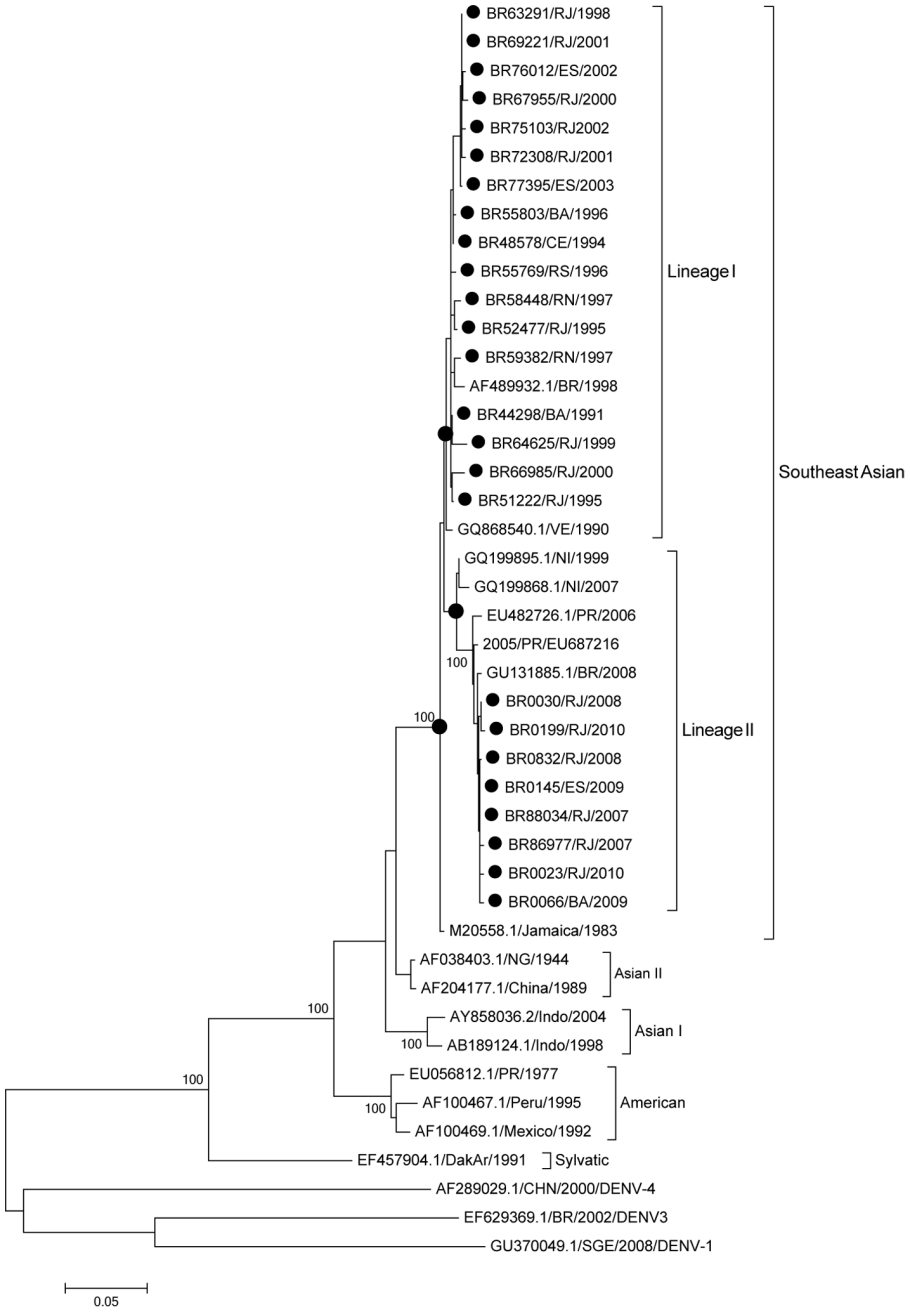
Maximum likelihood phylogeny based on the C/prM/M/E genes of 25 Brazilian DENV-2, 1991–2010. Black circles represent DENV-2 sequences generated in this study. Strains representative from the four genotypes available in Genbank (www.ncbi.nlm.nih.gov) were used for the comparison, DENV-1, DENV-3 and DENV-4 strains were used as outgroup to root the trees. The percentage of replicate trees in which the associated taxa clustered together in the bootstrap test (1000 replicates) is shown next to the branches. DENV strains used were named as follows: Country/strain number/state/year. RJ: Rio de Janeiro, ES: Espirito Santo, CE: Ceará, BA: Bahia, RS: Rio Grande do Sul, RN: Rio Grande do Norte.

**Figure 2:**
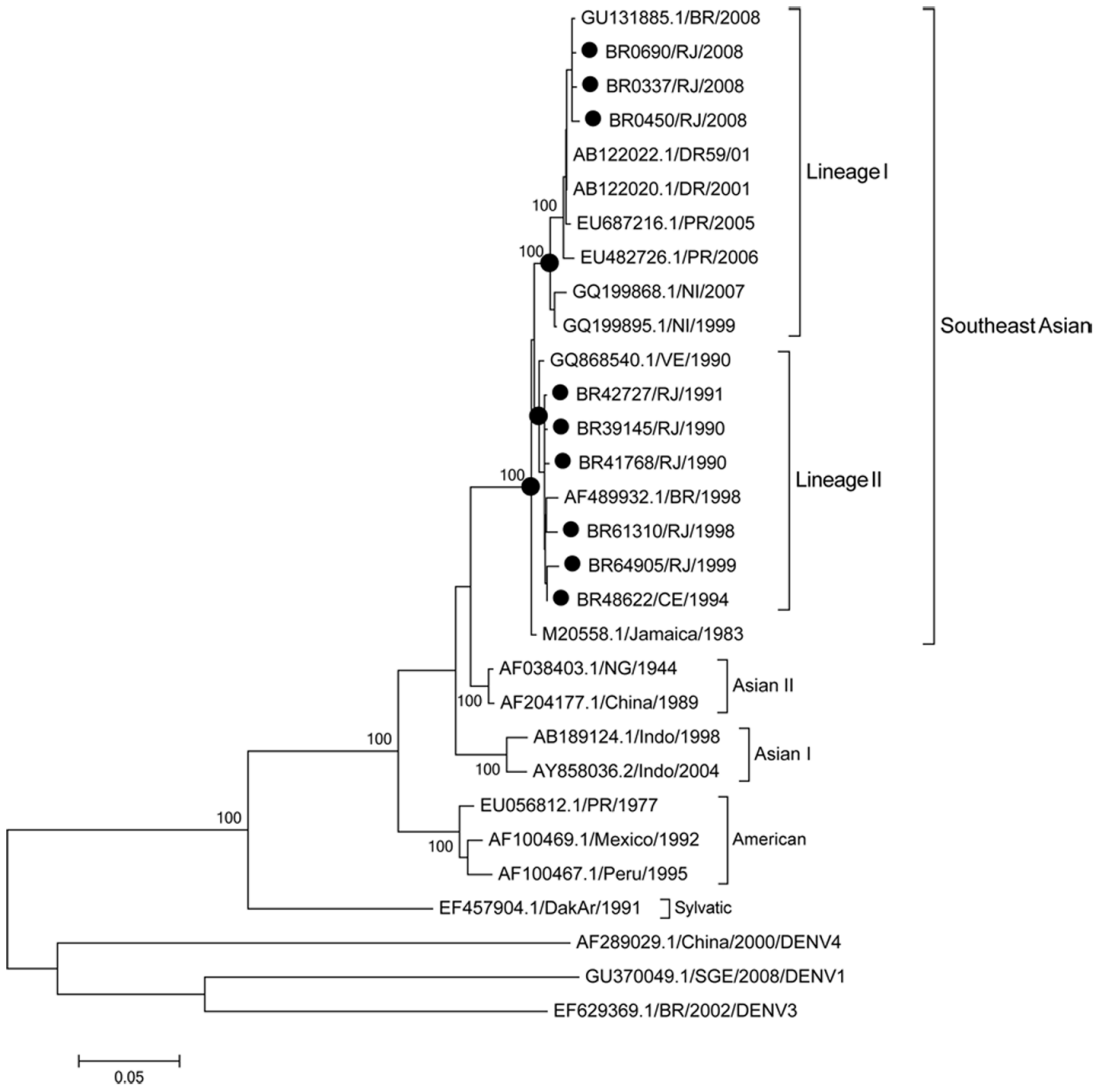
Maximum likelihood phylogeny based on the complete coding region sequencing of 9 Brazilian DENV-2, 1990–2008. Black circles represent DENV-2 sequences generated in this study. Strains representative from the four genotypes available in Genbank (www.ncbi.nlm.nih.gov) were used for the comparison, DENV-1, DENV-3 and DENV-4 strains were used as outgroup to root the trees. The percentage of replicate trees in which the associated taxa clustered together in the bootstrap test (1000 replicates) is shown next to the branches. DENV strains used were named as follows: Country/strain number/state/year. RJ: Rio de Janeiro and CE: Ceará.

In this study, the strains BR64022/98 isolated in the 90's and Jamaica 1983 were considered as reference strains for comparison purposes. The percentage of similarity among the 25 DENV-2 strains ranged from 80.3 to 99.9% when those compared to each other and to strains representative of the different genotypes available on GenBank. The partial genome sequencing analysis characterized the Brazilian DENV-2 strains from this study as belonging to the Southeast Asian genotype, however a distinction of two Lineages within this genotype has been identified. It was observed that strains circulating prior DENV-2 emergence (1990–2003) belong to Southeast Asian genotype, Lineage I and strains isolated after DENV-2 emergence in 2007 belong to Southeast Asian genotype, Lineage II (Figures 1 and 2). Furthermore, the latter were more closely related to strains from the Dominican Republic (DR59/01), representative from the Southeast Asian genotype, Lineage II.

When the 25 DENV-2 strains were compared to the strain BR64022/98, amino acid substitutions leading to change in the biochemical properties were observed on the C and prM genes. On the E gene, a total of twelve substitutions were observed, with nine resulting in a change on the amino acid change of biochemical property (Supplementary material 1). No consistent differences were observed on the E gene from strains isolated from cases with different clinical manifestations analyzed, suggesting that if the disease's severity has a genetic origin, it is not only due to the differences observed on the E gene.

To determine whether possible amino acids differences on other genes were related to disease severity, we fully analyzed (coding region) DENV-2 strains (*n* = 9), representative of DF cases isolated from 1990 to 1999 and strains isolated from fatal cases occurred after the DENV-2 re-emergence after 2007 until 2010. The strain 0450/2008, representative of the DENV-2 re-emergence isolated from a DF secondary case who evolved to death was fully sequenced and its comparison to the strain from the Dominican Republic (DR59/2001), representative of the DENV-2 re-emergence, showed 22 amino acid substitutions. Likewise, the strain 0690/2008 isolated from a DHF case occurred also during the re-emergence of DENV-2 had nine had amino acid substitutions when compared to the strain DR59/2001, with seven of those leading to amino acid biochemical property change (Table S1).

The DENV-2 strain 0337/2008 isolated from a newborn presenting a high anti-DENV IgG titer who evolved to death, infected probably due transplacental transmission as his mother was diagnosed with acute DENV infection, showed substitutions on NS2A, NS4A and NS5, which were shared with the other two strains isolated from fatal cases (Table S2). The results obtained by the DENV-2 full-length genome sequencing did not point out consistent differences related to a more severe disease.

A substitution on E_390_ (N→D) was reported as resulting in a reduction in viral replication in macrophages and dendritic cells [19] whereas E_390_ (D→N) resulted in enhanced replication, maturation and activation of macrophages, enhancement of the immune response with an increased production of cytokines, increased vascular permeability and consequently a greater chance of developing DHF [20].All DENV-2 strains analyzed presented an asparagine (N) in E_390_, previously identified as a probable genetic marker of virulence observed in DHF strains from Asian origin.

The percentage of identity of the re-emergent DENV-2 with the Dominican Republic strain isolated in 2001 combined to the percentage of divergence with the strains first introduced in the country in the 90's suggests that those viruses did not evolved locally but were due to a new viral Lineage introduction in the country from the Caribbean.

## Discussion

In the Americas, the first DENV-2 was isolated in 1953 in Trinidad [21] and the first DHF epidemic caused by this serotype occurred in Cuba in 1981 after the introduction of DENV-2 genotype originated in Southeast Asia [10], [22]. Epidemics studies showed that the DENV-2 introduced in Brazil, Colombia, Venezuela and Mexico had a common ancestor with isolates from Southeast Asia, suggesting the direct transmission from that region to the Americas [23].

In Brazil, the first DHF/DSS cases were reported after the DENV-2 introduction in Rio de Janeiro [13], [24], [25], which spread to other states in the country. Phylogenetic analysis of DENV-2 strains circulating at that time confirmed the genotype circulating in Southeast Asia [26], [27]. This observation was further corroborated in an extensive analysis of viruses from the states of Rio de Janeiro (1990 and 1995), Ceará (1994), Bahia (1994 and 1999), Maranhão (1996 and 1998), Mato Grosso (1997), Pará (1998), Rio Grande do Norte (1998), Paraíba (1999) Sergipe (1999), Espiríto Santo (1995 and 2000) and forty strains isolated in Pernambuco (1995–2002) [28], [29].

After seven years without activity in Brazil, DENV-2 re-emerged in April of 2007 in the state of Rio de Janeiro causing the more severe dengue epidemic in the country in 2008 [30], [31]. Phylogenetic analysis of DENV-2 circulating in 90's and after its re-emergence identified two distinct lineages within the Southeast Asian genotype [32].

In the present study, the analysis based on the sequencing of the C/prM/M/E genes (2,325 bp) from 25 DENV-2 Brazilian isolates divided those strains in two distinct groups, one formed by DENV-2 isolated from 1991 to 2003 and another with strains isolated from 2007 to 2010 following the re-emergence of this serotype in the country. Corroborating previous phylogeny [26]–[29] strains isolated from 1991to 2003 were classified as Southeast Asian genotype, Lineage I and presenting similarities with the Brazilian strain BR64022/98 and the strain Jamaica/83. However, the strains isolated between 2007 and 2010, showed higher similarity with the strain DR59/01, from the Dominican Republic, representing the Southeast Asian genotype, Lineage II, corroborating the analysis by Oliveira *et al* [32]. A study by Aquino *et al* [33] demonstrated that DENV-2 strains from Paraguay could also be grouped into two distinct lineages within the Southeast Asian genotype and suggested the introduction of a new lineage possibly associated a serotype shift from DENV-3 to DENV-2, as observed in Brazil in 2007 and 2008 [31].

The absence of DENV-2 circulation in the years prior to its re-emergence and the high similarity observed between those viruses and the strain isolated in the Dominican Republic in 2001, suggests the introduction of a new lineage of DENV-2 causing the 2008 epidemic in Brazil. Romano *et al* [34] also demonstrated that DENV-2 strains isolated in Sao Paulo State in 2010 were in a monophyletic group with the strains circulating in Rio de Janeiro in 2007 and 2008 and that those were closely related to strains isolated in Cuba and Dominican Republic, with a small genetic distance, suggesting that this new lineage of DENV-2 re-emerged in of Brazil may have been imported the Caribbean. Although genetic variants of DENV have been implicated in disease severity in the past [35], [36], it was with the advance of evolutionary studies based on phylogenetic analysis combined to epidemiological data that genotypes within the distinct serotypes were associated with a greater or lesser disease severity [11], [37]–[40].

The strain isolated from a DHF case in 2000 (strain RJ/67922/2000) presented an exclusive substitution on prM_143_ (T→I) when compared to the other strains analyzed in this study. However, substitutions related to DHF/DSS cases were identified on prM_16_ and prM_81_ [41].

Substitutions were found on the residues E_129_ (V→I) and E_131_ (L→Q), and these are related to the division of the Southeast Asian genotype in two distinct clades, corroborating the observations that amino acids on E_129_ and E_131_ are in critical markers for genetic classification of DENV [33], [42].

All 34 strains analyzed in this study presented an asparagine (N) on E_390_, previously characterized as a probable trigger for DHF detected in strains of Asian origin [43]. Mutations on the flaviviruses domain III of E protein can induce virulence or attenuation of the virus to escape from the immune system [44], [45] and in this study, changes were observed throughout this domain (aa 297 to 394). The DHF case, which culminated in death (59382/1997) showed amino acid differences only in the E gene, but those differences were shared with other DF cases strains, when they were compared to the strain BR64022/98.

In this study, a substitution on prM_39_ was observed on the strain 0690/2008 isolated from a DHF case with a fatal outcome, on the strain 55769/1996 from a DF case and on the strain 0199/2010.. Catteau *et al* [46] demonstrated that the intracellular production of M ectodomain of all four DENV serotypes of DENV induce apoptosis in host cells. The carboxy terminus of prM protein with nine amino acids (aa 32–40) of some flaviviruses was designated as Apopto M [46] and appears to play an important role in inducing apoptosis and cytopathic effects [46]–[48].

Several changes were observed along the NS protein genes. Studies conducted by Yábar, [49] show that mutations in NS1 are related to the development of DHF/DSS cases when they were compared to patients with DF.

Despite the functional importance of mutations in NS genes remains unknown, future studies can elucidate their role in the emergence of strains and/or pathogenesis of the disease. It was not possible to correlate the role of Lineage II emergence with an increased severity of cases observed in the period between the years 2007–2010. Furthermore, the occurrence of secondary infection may have been the risk factor for the development of more severe cases.

In conclusion, this result shows a temporal circulation of genetically different viruses in Brazil probably due to the introduction of a new viral lineage from the Caribbean which lead to the re-emergence of this serotype after 2007. In 2007–2008, DENV-2 was responsible for most severe epidemic already described in the country, with 787,726 cases reported and 491 deaths [31]. Moreover, the Caribbean has been suggested as an important region for the circulation of DENV-2, importation and exportation of strains from and to Central America and South America [42], [50], [51].

In the past 20 years, DENV-2 activity in Brazil has contributed significantly to changes in the disease morbidity and sudden age shift [30]. In dengue endemic countries, displacement of DENV serotypes, genotypes and lineages have been reported previously and have been associated with changes in the disease severity [40], [52]–[55]. This emphasizes the need of straightening virological surveillance to monitor the emergence or re-emergence of DENV strains with pathogenic potential to cause epidemics.

## Supporting Information

Table S1 **Molecular characterization of DENV-2 strains isolated in Brazil based on the partial genes analysis.**
(DOCX)

Table S2 **Molecular characterization of DENV-2 isolated in Brazil based on the complete coding region analysis.**
(DOCX)
