# Supplementary material for: Twenty Years of DENV-2 Activity in Brazil: Molecular Characterization and Phylogeny of Strains Isolated from 1990 to 2010
Source: PLoS Negl Trop Dis. 2013 Mar 14;7(3):e2095. doi: 10.1371/journal.pntd.0002095 (PMC3597488; doi:10.1371/journal.pntd.0002095)
Supplement: Table S2 — Molecular characterization of DENV-2 isolated in Brazil based on the complete coding region analysis. (DOCX) [file pntd.0002095.s002.docx]

Molecular characterization of Dengue virus type 2 (DENV-2) strains isolated in Brazil from 1990 to 2008 based on the complete genome (coding region) analysis.

| DENV-2 strains | Gene/Amino acid position | | | | | | | | | | | | | | | | | | | |
| --- | --- | --- | --- | --- | --- | --- | --- | --- | --- | --- | --- | --- | --- | --- | --- | --- | --- | --- | --- | --- |
|  | Clinical  Presentation | C | | | | | prM | | | | | | E | | | | | | | |
|  |  |  |  |  |  |  |  |  |  |  |  |  | Domain II | | | | | | | Domain I |
|  |  | 10 | 97 | 104 | 106 | 112 | 15 | 39 | 55 | 81 | 120 | 134 | | 71 | 91 | 126 | 129 | 131 | 160 | 170 |
| BR64022/1998* | DF | S | R | V | I | A | G | I | L | T | V | A | | E | I | E | V | L | E | I |
| Jamaica/1983 | - | . | . | . | . | . | . | . | . | A | . | T | | . | L | . | . | . | K | . |
| New Guinea/1944 | - | N | . | M | . | V | S | M | F | . | A | T | | D | V | K | . | Q | K | . |
| 39145/RJ/1990 | DF | . | . | . | . | . | . | . | . | . | . | . | | . | . | . | . | . | . | . |
| 41768/RJ/1990 | DF | . | . | . |  | . | . | . | . | . | . | . | | . | . | . | . | . | . | . |
| 42727/RJ/1991 | DF | . | . | . | . | . | . | . | . | . | . | . | | . | . | . | . | . | . | . |
| 48622/CE/1994 | DF | . | . | . | . | . | . | . | . | . | . | . | | . | . | . | . | . | . | . |
| 61310/RJ/1998 | DF | . | K | . | . | . | . | . | . | . | . | . | | . | . | . | . | . | K | . |
| 64905/RJ/1999 | DF | . | . | . | . | . | . | . | . | . | . | . | | . | . | . | . | . | K | . |
| 0337/RJ/2008 | Dead | . | . | . | V | . | . | . | . | . | . | T | | . | . | . | I | Q | K | T |
| 0450/RJ/2008 | DF/ Dead | . | . | . | V | . | . | . | . | . | . | T | | . | . | . | . | . | . | . |
| 0690/RJ/2008 | DHF/ Dead | . | . | . | V | . | . | M | . | . | . | T | | . | . | . | I | Q | K | T |
| DakAr/1991 | - | N | . | F | . | V | S | M | F | S | A | T | | . | V | . |  | Q | K | . |
| Mexico/1992 | - | N | . | M | . | V | S | M | F | . | A | T | | D | V | . | I | Q | K | . |
| DR59/2001* | - | . | . | . | V | . | . | . | . | . | . | T | | . | . | . | I | Q | K | T |

| DENV-2 strains | Gene/Amino acid position | | | | | | | | | | | | | | | | | |
| --- | --- | --- | --- | --- | --- | --- | --- | --- | --- | --- | --- | --- | --- | --- | --- | --- | --- | --- |
|  | Clinical Presentation | E | | | | | | | | | | | | NS1 | | | | |
|  |  | Domain II | Domain I | | Domain III | | | | Insoluble Region | | | | |  |  |  |  |  |
|  |  | 203 | 206 | 289 | 308 | 340 | 347 | 360 | 380 | 402 | 447 | 491 | 5 | | 105 | 208 | 212 | 261 |
| BR64022/1998* | DF | E | W | M | I | M | A | E | I | F | V | A | V | | R | D | I | Y |
| Jamaica/1983 | - | D | . | . | . | . | V | . | . | . | A | . | . | | . | . | M | H |
| New Guinea/1944 | - | N | . | . | V | . | V | . | . | I | A | V | . | | Q | . | M | H |
| 39145/RJ/1990 | DF | . | . | . | . | . | V | . | . | . | A | . | . | | . | . | M | H |
| 41768/RJ/1990 | DF | . | . | . | . | . | V | . | . | . | A | . | . | | . | . | M | H |
| 42727/RJ/1991 | DF | . | . | . | . | . | V | . | . | . | A | . | . | | . | . | M | H |
| 48622/CE/1994 | DF | . | . | . | . | . | V | . | . | . | A | . | . | | . | . | M | H |
| 61310/RJ/1998 | DF | . | . | L | . | . | V | G | . | . | A | . | . | | . | G | M | H |
| 64905/RJ/1999 | DF | . | . | . | . | . | V | . | . | . | A | . | . | | . | . | M | H |
| 0337/RJ/2008 | Dead | D | . | . | . | T | V | . | V | . | A | . | I | | . | . | M | H |
| 0450/RJ/2008 | DF/ Dead | D | . | . | . | T | V | . | V | . | A | . | I | | . | . | M | H |
| 0690/RJ/2008 | DHF/ Dead | D | . | . | . | T | V | . | V | . | A | . | I | | . | . | M | H |
| DakAr/1991 | - | S | . | . | V | . | V | . | . | . | A | V | . | | K | . | M | . |
| Mexico/1992 | - | D | . | . | . | . | V | . | . | . | A | V | . | | . | . | . | H |
| DR59/2001* | - | D | . | . | . | T | V | . | V | . | A | . | I | | . | . | M | H |

Cont

Cont

| DENV-2 strains | Gene/Amino acid position | | | | | | | | | | | | | | | | | | | | | | | |
| --- | --- | --- | --- | --- | --- | --- | --- | --- | --- | --- | --- | --- | --- | --- | --- | --- | --- | --- | --- | --- | --- | --- | --- | --- |
|  | Clinical Presentation | NS2A | | | | | | | | | | | | | | | | | | | NS2B | | | |
|  |  | 32 | 8 | 48 | 76 | 119 | 63 | 104 | 109 | 116 | 133 | 135 | 136 | 139 | 153 | 162 | 174 | 181 | 189 | 215 | 8 | 48 | 76 | 119 |
| BR64022/1998* | DF | V | V | S | I | V | T | A | A | I | V | K | I | N | S | L | A | L | T | S | V | S | I | V |
| Jamaica/1983 | - | A | I | . | . | . | . | . | . | . | . | . | . | . | L | Q | . | . | A | . | I | . | . | . |
| New Guinea/1944 | - | A | I | . | . | L | . | T | V | . | . | . | M | K | L | Q | V | F | A | N | I | . | . | L |
| 39145/RJ/1990 | DF | A | . | . | . | . | . | . | . | . | . | . | . | . | . | . | . | . | . | . | . | . | . | . |
| 41768/RJ/1990 | DF | A | . | . | . | . | . | . | . |  | . | . | . | . | . | . | . | . | . | . | . | . | . | . |
| 42727/RJ/1991 | DF | A | . | . | . | . | P | . | . | . | . | . | . | . | . | . | . | . | . | . | . | . | . | . |
| 48622/CE/1994 | DF | A | . | . | . | . | . | . | . | . | . | . | . | . | . | . | . | . | . | . | . | . | . | . |
| 61310/RJ/1998 | DF | A | . | . | . | . | A | . | T | . | . | . | . | . | . | . | . | . | . | . | . | . | . | . |
| 64905/RJ/1999 | DF | A | . | . | . | . | . | . | . | . | . | R | . | . | . | . | . | . | . | . | . | . | . | . |
| 0337/RJ/2008 | Dead | A | I | . | . | . | . | . | . | L | A | . | . | . | . | Q | . | . | A | . | I | . | . | . |
| 0450/RJ/2008 | DF/ Dead | A | I | . | V | . | . | . | . | L | A | . | . | . | . | Q | . | . | A | . | I | . | V | . |
| 0690/RJ/2008 | DHF/ Dead | A | I | A | . | . | . | . | . | L | A | . | . | . | . | Q | . | . | A | . | I | A | . | . |
| DakAr/1991 | - | A | I | . | V | L | A | . | V | . | . | . | . | S | L | Q | . | . | . | G | I | . | V | L |
| Mexico/1992 | - | A | I | . | . | L | . | T | V | . | . | . | M | . | L | Q | V | . | A | . | I | . | . | L |
| DR59/2001* | - | A | I | . | . | . | . | . | . | L | . | . | . | . | . | Q | . | . | A | . | I | . | . | . |

Cont

| DENV-2 strains | Gene/Amino acid position | | | | | | | | | | | | | | | | | | | | | |
| --- | --- | --- | --- | --- | --- | --- | --- | --- | --- | --- | --- | --- | --- | --- | --- | --- | --- | --- | --- | --- | --- | --- |
|  | Clinical  Presentation | NS3 | | | | | | | | | | | | | | | | | | | | |
|  |  | 15 | 29 | 62 | 116 | 121 | 141 | 143 | 183 | 187 | 188 | 250 | 290 | 346 | 396 | 400 | 419 | 462 | 467 | 550 | 562 | 607 |
| BR64022/1998* | DF | E | R | R | L | T | V | R | F | K | R | T | T | N | V | T | R | I | R | R | R | A |
| Jamaica/1983 | - | G | . | K | . | . | . | . | I | . | . | A | . | . | . | A | K | . | K | . | . | . |
| New Guinea/1944 | - | G | K | K | . | A | I | K | I | R | K | A | . | S | . | . | K | . | K | . | . | T |
| 39145/RJ/1990 | DF | . | . | . | . | . | . | . | I | . | . | A | . | . | . | A | . | . | K | . | . | . |
| 41768/RJ/1990 | DF | G | . | . | . | . | . | . | I | . | . | A | . | . | . | A | . | . | K | . | . | . |
| 42727/RJ/1991 | DF | G | . | . | . | . | . | . | I | . | . | A | . | . | . | A | . | . | K | . | . | . |
| 48622/CE/1994 | DF | . | . | . | . | . | . | . | I | . | . | A | . | . | . | A | . | . | K | . | . | . |
| 61310/RJ/1998 | DF | G | . | . | . | . | . | . | I | . | . | A | . | . | . | A | . | V | K | . | . | . |
| 64905/RJ/1999 | DF | . | . | . | . | . | . | . | I | . | . | A | . | . | . | A | . | . | K | . | . | . |
| 0337/RJ/2008 | Dead | G | . | . | I | . | . | . | I | . | . | A | . | . | . | A | K | . | K | K | K | . |
| 0450/RJ/2008 | DF/ Dead | G | . | . | I | . | . | . | I | . | . | A | S | . | . | A | K | . | K | K | K | . |
| 0690/RJ/2008 | DHF/ Dead | G | . | . | I | . | . | . | I | . | . | A | . | . | A | A | K | . | K | K | K | . |
| DakAr/1991 | - | G | . | K | . | . | I | K | I | . | . | A | . | . | T | . | K | . | K | K | K | . |
| Mexico/1992 | - | G | K | K | . | . | . | K | I | R | . | A | . | . | . | . | K | . | . | K | . | . |
| DR59/2001* | - | G | . | . | I | . | . | . | I | . | . | A | . | . | . | A | K | . | K | K | K | . |

| DENV-2 strains | Gene/Amino acid position | | | | | | | | | | | | | | | | | |
| --- | --- | --- | --- | --- | --- | --- | --- | --- | --- | --- | --- | --- | --- | --- | --- | --- | --- | --- |
|  | NS4A | | | | | | | NS4B | | | | | | | | | | |
|  | 23 | 36 | 39 | 42 | 46 | 63 | 76 | 12 | 15 | 22 | 23 | 57 | 64 | 87 | 88 | 112 | 156 | 175 |
| BR64022/1998* | D | A | R | N | S | T | K | D | L | E | S | E | S | M | D | L | E | I |
| Jamaica/1983 | . | . | . | . | . | . | . | . | . | . | . | . | . | I | H | . | D | . |
| New Guinea/1944 | . | . | . | . | . | . | R | . | . | Q | P | . | . | . | . | F | D | V |
| 39145/RJ/1990 | . | . | . | . | . | . | . | . | . | . | . | . | . | . | . | . | . | . |
| 41768/RJ/1990 | . | . | . | . | . | . | . | . | . | . | . | . | . | . | . | . | . | . |
| 42727/RJ/1991 | . | . | . | . | . | . | . | . | . | . | . | . | . | . | . | . | . | . |
| 48622/CE/1994 | . | . | . | . | . | . | . | . | . | . | . | . | . | . | . | . | . | . |
| 61310/RJ/1998 | . | . | . | . | . | . | . | . | . | . | . | . | . | . | . | . | . | . |
| 64905/RJ/1999 | . | . | . | K | . | . | . | . | F | . | . | . | . | . | . | . | . | . |
| 0337/RJ/2008 | N | V | K | T | . | A | . | . | . | . | . | . | . | . | . | . | . | . |
| 0450/RJ/2008 | N | V | K | T | N | A | . | Y | F | . | . | V | T | . | . | . | . | . |
| 0690/RJ/2008 | N | V | K | T | . | A | . | . | . | . | . | . | . | . | . | . | . | . |
| DakAr/1991 | . | M | . | . | . | . | . | . | . | Q | P | . | . | . | . | . | . | V |
| Mexico/1992 |  | . | K | . | . | . | . | . | . | Q | P | . | . | . | . | . | D | V |
| DR59/2001* | N | V | . | . | . | . | . | . | F | . | . | . | . | . | . | . | . | . |

Cont

| DENV-2 strains | Gene/Amino acid position | | | | | | | | | | | | | | | | | | |
| --- | --- | --- | --- | --- | --- | --- | --- | --- | --- | --- | --- | --- | --- | --- | --- | --- | --- | --- | --- |
|  | NS5 | | | | | | | | | | | | | | | | | | |
|  | 5 | 19 | 23 | 156 | 168 | 196 | 225 | 246 | 254 | 341 | 375 | 388 | 412 | 429 | 437 | 501 | 514 | 521 | 523 |
| BR64022/1998* | I | T | N | I | A | T | T | K | P | V | R | K | I | S | R | E | R | D | S |
| Jamaica/1983 | . | A | S | . | V | . | S | R | T | M | K | . | . | . | . | G | K |  | . |
| New Guinea/1944 | . | A | S | V | V | A | S | . | . | M | K | . | . | . | K | . | K | . | . |
| 39145/RJ/1990 | . | A | S | . | V | . | . | . | . | M | . | . | . | . | . | G | . | . | . |
| 41768/RJ/1990 | . | A | S | . | V | . | . | . | . | M | . | . | . | . | . | G | . | . | . |
| 42727/RJ/1991 | . | A | S | . | V | . | . | . | . | M | . | . | . | . | . | G | . | . | . |
| 48622/CE/1994 | . | A | S | . | . | . | . | . | . | M | . | . | . | . | . | G | . | . | . |
| 61310/RJ/1998 | . | A | S | . | V | . | . | . | . | M | . | . | . | . | . | G | . | . | . |
| 64905/RJ/1999 | . | A | S | . | . | . | . | . | . | M | . | . | . | . | . | G | . | . | . |
| 0337/RJ/2008 | V | A | S | . | V | . | . | . | . | M | K | E | V | G | . | G | . | E | G |
| 0450/RJ/2008 | V | A | . | . | . | . | . | . | . | M | K | E | V | G | . | . | . | . | . |
| 0690/RJ/2008 | V | A | S | . | V | . | . | . | . | M | K | E | V | G | . | G | . | E | G |
| DakAr/1991 | . | A | S | V | V | A | S | R | . | M | . | R | V | E | K | . | K | . | . |
| Mexico/1992 | . | A | S | V | V | . | S | R | . | M | K | . | . | N | K | . | K | E | . |
| DR59/2001* | V | A | S | . | V | . | . | . | . | M | K | E | . | G | . | G | . | E | G |

Cont

| DENV-2 strains | Gene/Amino acid position | | | | | | | | | | | |
| --- | --- | --- | --- | --- | --- | --- | --- | --- | --- | --- | --- | --- |
|  | NS5 | | | | | | | | | | | |
|  | 558 | 596 | 628 | 637 | 670 | 717 | 749 | 763 | 811 | 825 | 836 | 878 |
| BR64022/1998* | K | R | I | V | L | V | K | T | A | E | V | E |
| Jamaica/1983 | E | . | . | . | . | . | . | . | . | . | . | . |
| New Guinea/1944 | E | . | V | . | . | I | R | S | T | . | P | . |
| 39145/RJ/1990 | . | . | . | . | . | . | . | . | . | . | . | . |
| 41768/RJ/1990 | . | . | . | . | . | . | . | . | . | . | . | . |
| 42727/RJ/1991 | . | . | . | . | . | . | . | . | . | . | . | . |
| 48622/CE/1994 | . | . | . | . | . | . | . | . | . | . | . | . |
| 61310/RJ/1998 | . | . | . | . | . | . | . | . | . | G | . | . |
| 64905/RJ/1999 | . | K | . | . | . | . | . | . | . | . | . | . |
| 0337/RJ/2008 | . | K | . | A | I | . | . | . | . | . | . | G |
| 0450/RJ/2008 | . | . | . | A | I | . | . | . | . | . | I | G |
| 0690/RJ/2008 | . | K | . | A | I | . | . | . | . | . | . | G |
| DakAr/1991 | E | . | L | P | . | I | R | S | . | . | I | . |
| Mexico/1992 | A | . | . | A | . | I | R | S | T | . | I | . |
| DR59/2001* | . | K | . | A | I | . | . | . | . | . | . | . |

Cont.

Bahia, CE: Ceara, RJ: Rio de Janeiro, RS: Rio Grande do Sul, RN: Rio Grande do Norte, ES: Espírito Santo, DR: Dominican Republic; C: capsid, prM/M: pre-membrane/membrane, E: envelope; DF:dengue fever, DHF:dengue haemorrhagic fever, DSS: Dengue Shock Syndrome; S: serine, R: Arginine, V: valine, I: isoleucine, A: alanine, G: glycine, D:aspartic acid, L: leucine, T: threonine, M: methionine, F: phenylalannine, K:lysine, Q: glutamine, N: asparagine. Dark gray block: DENV-2 strains from 1991 to 2003, light Gray area: DENV-2 strains, *: Strains used for comparison purposes; Dot (.): indicates similarity.
